# Opportunities to learn and barriers to change: crack cocaine use in the Downtown Eastside of Vancouver

**DOI:** 10.1186/1477-7517-5-34

**Published:** 2008-11-17

**Authors:** Susan Boyd, Joy L Johnson, Barbara Moffat

**Affiliations:** 1 Studies in Policy & Practice, University of Victoria, Canada; 2 NEXUS, School of Nursing, University of British Colombia, Canada; 3 Nursing and Health Behaviour Research Unit, School of Nursing, University of British Columbia, Canada

## Abstract

In 2004, a team comprised of researchers and service providers launched the Safer Crack Use, Outreach, Research and Education (SCORE) project in the Downtown Eastside of Vancouver, British Columbia, Canada. The project was aimed at developing a better understanding of the harms associated with crack cocaine smoking and determining the feasibility of introducing specific harm reduction strategies. Specifically, in partnership with the community, we constructed and distributed kits that contained harm reduction materials. We were particularly interested in understanding what people thought of these kits and how the kits contents were used. To obtain this information, we conducted 27 interviews with women and men who used crack cocaine and received safer crack kits. Four broad themes were generated from the data: 1) the context of crack use practices; 2) learning/transmission of harm reducon education; 3) changing practice; 4) barriers to change. This project suggests that harm reduction education is most successful when it is informed by current practices with crack use. In addition it is most effectively delivered through informal interactions with people who use crack and includes repeated demonstrations of harm reduction equipment by peers and outreach workers. This paper also suggests that barriers to harm reduction are systemic: lack of safe housing and private space shape crack use practices.

## 

In 2004, a team comprised of researchers and service providers launched the Safer Crack Use, Outreach, Research and Education (SCORE) project in the Downtown Eastside (DTES) of Vancouver, British Columbia, Canada. The purpose of the SCORE project was to develop a better understanding of the harms associated with crack cocaine smoking and to determine the feasibility of specific harm reduction strategies. A significant harm reduction component of the project included the distribution of safer crack kits. The findings for this paper are derived from 27 qualitative interviews conducted in 2007 with women and men who use crack cocaine and who had received safer crack kits.

The SCORE project was ultimately aimed at providing harm reduction services to women and men. The project was dedicated to ensuring that everyone who uses crack and lives in the DTES has access to equipment and education that will help them to incorporate safer crack use practices. At the outset, one of the many questions we had related to how crack cocaine users access and utilize educational information about illegal drug use and harm reduction. We were also interested in knowing how crack cocaine users themselves reduce drug-related harm.

SCORE drew from critical, feminist and harm reduction research on illegal drug use in constructing the research project and analyzing the research data. The project was also informed by community-based research perspectives. The following sections include background on critical and feminist drug research, harm reduction perspectives, methodology and project background, safer crack kits and distribution, and research findings. We conclude by highlighting social factors that shape crack use and learning opportunities. The project findings contribute to existing harm reduction literature and are expected to be of benefit to practitioners working in the area of harm reduction.

### Critical and Feminist Drug Research

Early critical drug research shifted the field of drug research. Rather than law officers or social workers, health professionals, or media people reporting on the lives of people who use illegal drugs, critical drug researchers adopted an ethnographic approach – privileging the voice and perspective of people who use drugs themselves in order to better understand their behaviour and concerns [1,2]. Later feminist ethnographic research explored how illegal drug use is gendered [3]. Critical and feminist drug research is especially useful for those seeking to understand social and cultural factors that shape the lives of people who use legal and illegal drugs. It privileges the subjective experiences of people who use drugs and provides insight into social learning related to minimizing harm and informal social control.

Most significant for this paper, critical and feminist drug researchers emphasize qualitative interviews as a methodological tool that successfully brings to light how people who use drugs learn and make sense of the drugs they consume and the social environment where they use them. Howard Becker's early 1963 ethnography makes clear that drug use and learning is shaped by sociohistorical, cultural, and social-psychological variables [2]. Drug researcher Norman Zinberg outlined how "set and setting" shape drug use. "Set," comprised of people's attitudes and their expectations, can be just as important, or more important, than the pharmacology of a drug in shaping a user's long-term relationship with a particular drug. "Setting" refers to the physical, cultural, and social environment in which a drug is used. These variables are not separate and distinct; rather they interact with one another [4].

Zinberg explained that the experience of drug use is also shaped by both formal (the law) and informal social controls, rituals, and the transmission of knowledge. He investigated how informal knowledge is transmitted, stating that it is a "crucial factor in the controlled use of any intoxicant" [4] (p. 14). Zinberg argued that rituals and informal controls provide opportunities for learning how to control the consumption of legal and illegal drugs and techniques of use and knowledge about equipment and safety. Informal controls apply to all drug use, for example, "don't drink till 5" and "don't bogart that joint" are familiar refrains. In addition to the concept of informal rituals and social control mechanisms that people who use drugs employ, drug researchers note that drug availability and access to drug paraphernalia (such as needle exchange) have a significant impact, especially on the lives of people who use illegal drugs, as do the criminal sanctions that shape drug use and services [1,5-9]. These concerns also shape the lives of people who use crack in the DTES.

In their extensive ethnography, which included 267 life-history interviews with heavy cocaine users (including crack) in northern California, Waldorf, Reinarman, and Murphy found that for these users "there is no central clearinghouse for such illicit information .... and crack users are left to their own folk-experimental devices for testing tools or techniques" [10] (p. 113). They also discovered that there was no "uniform progression or pattern" of cocaine use and that their participants were by and large no different than other "ordinary citizen [s]" (i.e., who held jobs and have families); this "normality" "turns out to be theoretically crucial" [10] (p. 10). They concluded that what keeps "many heavy users from falling into the abyss of abuse, and what helps pull back those who do fall, is precisely this *stake in conventional life*" [10] (italics in original, p. 10).

Critical and feminist drug researchers have long pointed out that there is much "historical evidence suggesting that reducing harm and offense...is more likely through the dissemination and internalization of the informal social controls of user culture than through the formal social controls of the state [11] (p. 408). Thus, it is imperative to understand the user culture and to work with them alongside community organizations in order to create better educational material, avenues for education, and access to harm reduction equipment. This was very much in keeping with the vision of the SCORE project.

### Harm Reduction

Although there are some different views about harm reduction, for example some conventional critics have co-opted the term to include sending people who use illegal drugs to prison to reduce risk to themselves and society, for the purposes of this paper, "harm reduction" is defined as providing practical, non-judgmental services that seek to minimize drug-related harm to both the individual and society. In addition, prohibition and social and economic inequality are seen as contributing to harm [6,8]. Harm reduction advocates state that " [a]dverse health, social and economic consequences of drug use" can be effectively decreased without "requiring decrease in drug use" [12] (p. 1698). Diverse, low, and high threshold services that are culturally appropriate have been created in and outside of Canada [13]. Prioritizing of needs and education are also central goals. Harm reduction programs differ from conventional abstinence-based treatment. Abstinence is not the primary goal of the harm reduction model; rather, abstinence is one of many varied options that can be offered. Drug use is understood as being non-static and can range to include a variety of methods of expression, including casual, dependent, functional, controlled, and dependent use. Furthermore, not all drug use is negative, nor is all addiction negative. Problematic or negative use is recognized as stemming from social factors and individual trauma; however, this is neither determined nor static [14].

Responding to political and social factors, and shifts in illegal drug availability and use, people who use illegal drug users themselves, health, and service providers continue to adapt their services to meet the needs of those most affected. Harm reduction advocates recognize the importance of moving towards a health and human rights model where social factors are brought to the foreground [15]. Furthermore, people who use illegal drugs are considered to be essential partners and experts at all levels of planning and practice [16]. Thus, we adopted a bottom-up approach to our harm reduction project, and members of the SCORE team worked in partnership with individuals who used crack [8].

In many ways, critical drug research perspectives and harm reduction practice draw on similar theoretical philosophy. Both perspectives recognize that those most affected by policy, and engaged in drug use, have insights to share about their lives. Both perspectives are interested in people's relationships with drugs and are non-judgmental about drug use in itself. Furthermore, both perspectives account for the social and cultural context in which drug use occurs. And finally, both perspectives recognize that humans have historically used drugs to change consciousness and that "zero tolerance" or a "drug-free" world is impossible and unrealistic. In this way, drugs are neither seen as bad nor good; rather, people use drugs for a variety of reasons – most often drugs are consumed for pleasure, to heal and sustain health, and for spiritual and religious reasons. However, people also use drugs to enhance physical performance, work, and school output; to alleviate hunger; to reduce pain and suffering (both physical and emotional); and as a strategy to cope with violence, dislocation, and colonization [6,14,17]. Critical drug research and harm reduction studies attempt to highlight how drug use is shaped by personal and larger societal and cultural factors.

### Harm Reduction in the DTES

In the late 1990s, Canada had methadone maintenance programs and needle-exchange services in place in some cities, now referred to as harm reduction programs; however, these services were less effective than they could have been due to one-on-one needle exchange and limited access and hours. In the case of methadone, punitive and ever-changing policy also limited its effectiveness [5,18]. Following years of advocacy by health and community groups including activists, especially by VANDU and the release of the *Report on the Task Force Into Illicit Narcotic Overdose Deaths in British Columbia *[19], a public health emergency was declared in 1997 by Dr. John Blatherwick, the Chief Medical Health Officer of the Vancouver Richmond Health Board, in response to increasing numbers of overdose deaths and infection for Hepatitis C and HIV in the area [20]. In 2001, the City of Vancouver's drug strategy, described in *A Framework for Action: A Four-Pillar Approach to Drug Problems in Vancouver*, was adopted by City Council [21]. The City drug strategy recommended actions across the four pillars of prevention, treatment, harm reduction, and enforcement. It also recommended the opening of the first supervised safer injection site in North America in the DTES. The facility, Insite, opened in 2003.

Programming that supports safer injection drug use practices such as access to sterile syringes and water and the implementation of a supervised injection site have been implemented in Vancouver [21]. In the DTES, harm reduction program planning has primarily focused on injecting drug use and the reduction of blood-borne infectious diseases such as HIV and Hepatitis C. Nevertheless, various infectious diseases have been associated with crack use. The scarcity of quality crack pipes (such as Pyrex pipes, which are more heat resistant and less likely to crack than glass pipes) and their cost leads to repeated use of glass pipes that are cracked and split. Split and cracked pipes increase the likelihood of cuts to the hands and lips [22-24]. In addition, many people who smoke crack share their equipment, thereby increasing their risk for infection [24-26]. Small and Drucker outlined some of the health risks that individuals who use crack are exposed to due to inadequate harm reduction equipment [9]. Among other risks, they noted how inadequate filters, such as Brillo, pose health risks to people who use crack because particles break off, putting users at risk for cuts to their lips as well as associated pulmonary problems [9,27].

There is also evidence of infections related to crack use. Hepatitis C (HCV) and Human immunodeficiency virus (HIV) have been associated with crack use in epidemiologic studies [23,28-31]. A recent study confirmed the plausibility of HCV transmission through sharing crack pipes when HCV was identified on a crack pipe [32]. In addition, a recent outbreak of pneumococcal pneumonia was identified in the DTES [33,34]. A substantial proportion of these cases were noted to be people who were using crack regularly, leading to the proposition that sharing crack paraphernalia was an efficient means of spreading pneumonia. The pneumonia outbreak generated high use of intensive care beds in the city, significant mortality, and a massive vaccination program [34]. The extent of the outbreak illustrated the precarious health status of people in the DTES or in contact with this community. In 2007 and 2008, there were also outbreaks of tuberculosis in persons who use crack in BC [33,35].

Originally a safer crack cocaine smoking room was planned for the Insite project; however, to date it has not been implemented. Few services provide support uniquely for people who use crack, and there is less harm reduction information, education, services, and access to safe equipment in the DTES [9,25]. Furthermore, "poverty, violence, exploitation, discrimination," and "ongoing trauma" intersect with and influence health concerns experienced by individuals who use crack in the DTES, especially women [33]. Of note, a 2006 SCORE survey (of 126 women and 80 men) conducted prior to kit construction and distribution in the DTES suggested a high incidence of daily and weekly crack smoking practices, use of Brillo (98.4%), split pipes (43.7%), and sharing pipes (46.8%) [25]. The findings reinforced the need for less harmful non-injection drug-using equipment, including Pyrex stems, metal screens, mouthpieces, and wooden push sticks, as well as further exploration of learning opportunities and barriers to change that resulted from kit distribution. The qualitative interviews were an attempt to better understand this from the perspective of those who had received safer crack kits.

## Methodology and background

The SCORE project was conducted in the DTES of Vancouver, British Columbia. It is one of the poorest neighbourhoods in Canada. It has been estimated that approximately 16,000 people live in the DTES and that women comprise 38% of this population [36]. The DTES is a very diverse neighbourhood: 40% of its residents are Aboriginal, and another 20% are East Asian or Latino/a [37]. The DTES has a "high concentration of social problems, including poverty, mental illness, drug use, crime, survival sex work, high HIV/Hepatitis infection rates, unemployment and violence" [38] (p. 5). A number of surveys in the DTES indicated that crack use has become increasingly common over the past 10 years. The actual prevalence of use depends on the population surveyed. In 2003–2004, the Community Health and Safety Evaluation project [39] recruited over 3,500 people within the DTES to participate in a survey on health-related questions. About 28% reported frequent crack use, and over 50% had used crack [39]. In a study of youth in custody in BC aged 14–19 in 2006, 60% reported ever using crack, with females significantly more likely than males to have used [40]. In addition, the Vancouver Injection Drug User Survey (VIDUS) found that crack use in a group of injection drug users in Vancouver almost doubled from 32% in 1997 to over 60% in 2004 [41]

The project drew from community-based research perspectives that aim to create social change and to give back to the community [42]. This methodological approach takes into account the lives of those who are acted upon – without erasing their experiences. A number of critical and feminist researchers advance community-based research as an approach that acknowledges that research participants are sources of knowledge about themselves and their communities; therefore, they have much to contribute [43,44]. The 2005 Canadian HIV/AIDS Legal Network paper, "*Nothing about us without us." Greater, meaningful involvement for people who use drugs: A public health, ethical and human rights imperative*," provided important guidance for inclusion of people who use illegal drugs [16].

The team was comprised of DTES community workers, research assistants, and faculty with backgrounds in nursing, health care and epidemiology, criminology/sociology. The team worked in collaboration with the Safer Crack Use Coalition of Vancouver (SCUC), a group comprised of community outreach workers, women and men who used crack cocaine, and health care providers, as well as the SCORE's Women's Advisory Committee (SWAC), comprised of four women selected from a women's support group run by the Vancouver Area Network of Drug Users (VANDU). Our intention was to provide an alternative model of research by including community input from the conception, to the planning, implementation, and writing about the project [25].

### Methods

Although major components of the SCORE project focused on women, men were also participants. The research activities included participant observation over a three-year period, field notes during the kit-making sessions, and cross-sectional surveys regarding health concerns and general drug use practices. These research activities surrounded the construction and distribution of non-injection harm reduction kits for crack use. Towards the end of the project, qualitative interviews were conducted.

The sample for this paper includes 27 qualitative interviews with women and men who use crack cocaine and who had received a harm reduction kit (17 women, 1 transgendered person, and 9 men). All interviews took place in the DTES. Interviews ranged from 15 to 45 minutes in length. The participants were between 19 to 55 years of age. Most of the women and men interviewed for the study were living in extreme poverty and currently used or had a recent history with crack use. Interviewees were recruited by SCORE members and staff from various locations throughout the DTES, including drop-in centres, women's housing facilities, emergency shelters, and community health centres. The drop-in locations were chosen strategically to enhance women's and men's access to services offered by these agencies. Participants were paid a $10 honorarium for their time and expertise, in keeping with practices of other health and social science research in the DTES and elsewhere; all were offered a safer crack kit.

The team developed an interview guide in which participants were asked about the first time they had received safer crack kits, the contents of the kits (what worked, what did not), changing crack use practices, and accessing crack kits. The questions were open-ended, and participants were encouraged to identify issues that they believed were relevant to their experience with safer crack kits and crack use.

Prior to the interview, interviewers reviewed the consent form with participants, and issues surrounding confidentially and anonymity were communicated. Recorded interviews were transcribed, and all identifying information was removed; transcribed interviews were then reviewed by the research team and coded. The transcripts were analyzed drawing on a method of constant comparison and questioning, a bottom-up, back-and-forth reflective process where "interpretation" informs the research process, including the coding process; thus, themes were identified not only through the interview schedule but from the data, and interviewing ceased upon reaching saturation [45,46].

### Safer Crack Kits

After consulting other harm reduction programs in Canada regarding the contents in safer crack kits, the SCORE team chose to include in each kit the items listed below. Decisions about the type and quantity of items took place through a process of consultation with people who use crack in Vancouver, as well as members of the project advisory teams, SCUC, and SWAC. The rationale for providing each of these items follows below. In total, approximately 14,000 kits were assembled during kit-making sessions (see Table 1).

**Table 1:** Kit contents and rationale for inclusion

***Kit Item***	***Rationale for Inclusion***
Pyrex Stems	• Compared to conventional glass, they are stronger and less brittle.
	• They are less likely to explode, break, or chip and last longer than do glass stems.
	• Their inclusion reduces likelihood of the use of other, less safe options.
Mouthpieces (a four-inch rubber tube)	• For use at one end of a stem to prevent direct contact with broken or hot pipes.
	• A personal mouthpiece minimizes exposure to communicable disease when a pipe is shared.
Wooden Push Sticks (chopstick)	• For the purpose of packing and positioning the filter or screen inside the stem.
	• Wooden push sticks do not chip stems, unlike metal counterparts that are used frequently (e.g., coat hangers, car antenna).
	• Given that plungers of syringes were also being used for this purpose, providing a wooden push stick decreased the use of syringes and subsequent littering of needles and syringes.
Condoms	• Since crack use is associated with high-risk sexual behaviors (i.e., buying and selling sex), condoms are integral to promoting safer sex.
	• Many women in the DTES who use crack support themselves through sex work; women need easy access to condoms.
Bandages:	• These were included to protect broken skin, promote healing, and minimize exposure to infection (self and others).
Alcohol Swabs	• Promoted the use of clean equipment (e.g., pipes, mouthpieces) and a means of cleaning wounds.
Screens (Brass tobacco pipe screens)	• They are less likely to break apart than steel wool or "Brillo.^1^"
	• Unlike Brillo, brass filters are not coated with potentially toxic substances.
Lighter	• Smoking crack requires applying consistent heat to the pipe.
	• Using matches is more likely to result in burns and the inhalation of sulphur.
Information cards	• Two cards were included in the kits: 1) The Tip card covered harm reduction information for people who use crack, and 2) The Resource card outlined information on health and drug services in the DTES for people who use drugs.

^1 ^The term "Brillo" used here and in the remainder of the document is the street term for the steel wool used as a filter on the inside of the crack pipe.

Over the course of the project, the kits were distributed through peer-delivered on-foot outreach or through an existing outreach van to persons in the DTES. All of the outreach teams distributed between 25 and 100 kits each shift. The process of distribution included handing out kits, demonstrating how to put the brass screens into the pipe and how to attach the mouthpiece properly. There was information provided on why screens should be used instead of Brillo. The teams also talked with people about the risks of sharing equipment and made referrals to health agencies when possible. The teams used a tally sheet to record how many kits were given out, the number of people who received demonstrations and education, what referrals were made, and the gender of kit recipients. The one-on-one interviews following kit distribution provide insight into crack cocaine practices among some individuals in the DTES of Vancouver.

## The findings

The findings point to the many challenges inherent in providing education and changing drug use practices among individuals who use crack. This was particularly the case for people who had a long history with crack use. Based on the interview data, we describe key findings, beginning with a description of the context of crack use.

### Crack use practices

The interview data revealed that the ways crack was smoked was shaped by the realities of people's lives. In order to understand how the kits were used, we must first consider this context. For example, many participants indicated that they often smoked in small groups and that this often necessitated the sharing of equipment, (i.e., a pipe). Due to a lack of private space and safe housing, the location for crack use for most participants was outdoors, e.g., on the streets and in alleys. One female participant noted:

*Because a lot of the time if women are on the street and they just want to have a toke and warm up*.

As a result of smoking crack in such open and public spaces, many talked about the need to be vigilant in order to avoid the police, which further contributed to a need to smoke crack in a hurry. One participant focused on the importance of being discrete with paraphernalia and remaining vigilant in her surroundings. She was particularly concerned that the push stick in the kit was too long:

It [the push stick] is a bit long for somebody who's trying to keep things out of sight. I noticed that if I am transporting some paraphernalia from one place to another and I have to get there fast and I don't want anybody noticing, I don't want the police to notice this stick hanging out of my pocket, it gets seen, right?

Most of the people we interviewed also spoke about *needing *to get high and being in a hurry to do so. Continuing to use Brillo for many was based on the belief that Brillo was easier to handle especially when in need of a "hoot." As one person stated, *"We're not thinking about safety when we want [to use], we're just thinking about our dope. We need a toke." *There was a particular concern that using screens would be awkward and would disrupt preferred practices. One woman indicated that she was in too much of a hurry when she was trying to get high and stated, "*I didn't want to play with it*" [inserting screens]. The context of people's day-to-day lives necessitated some degree of adaptability. Participants described how crack use practices varied according to circumstances. They often illustrated a practical approach to their use. In the following quote one participant describes the utility of sharing and why an extra mouthpiece is a good idea especially for street involved women:

*The two mouthpieces is really good because then they can keep one to use if people want to use their pipe, and they do lend it out because a lot of women don't have a lot of money. And if somebody is using their pipe, they get to keep their resin, and that's how they stay high all day, right? So if they lend their pipe out all day long and have an extra mouthpiece to put on for other people to use, then they can switch mouthpieces. I think that's a great idea*.

Their ability to adapt the use of the harm reduction materials was noteworthy. One woman described how she used condoms for smoking crack.

*You know how people share it when they're mouth to mouth blowing the smoke in, it's the same thing with a condom. You blow the smoke in there and suck it back. Same thing, "seconds," that's what I use the condoms for*.

While many did not consistently use harm reduction approaches when they smoked crack, their practices suggested an underlying concern about limiting harms. For example, one participant commented:

*I don't use the mouthpiece, if I do, if I'm using somebody else's [pipe], then I use a mouthpiece*.

We also found, not surprisingly, marginalization shaped crack use and learning opportunities. In particular, a lack of private space (affordable housing) and visibility shaped crack use and the experiences of participants in the DTES. As noted, the participants in this sample often smoked crack outside and in small groups. Sharing of pipes is common, and safety concerns related to violence, fear of arrest, and rip-offs keep users on the move. It follows that equipment that is time-consuming to use and difficult to work with remains a challenge to promote when "time" is a rare commodity. A sense of urgency to use crack set in the context of a lack of private space to use and busy days filled with volunteer work, participation in projects for research stipends, doctors' appointments, hustles to obtain drugs, food, and shelter limit learning opportunities and encounters with safer crack kit distribution teams.

### Learning/Transmission of harm reduction education

In this section of the findings, we explore the ways in which educational information was conveyed that resulted in learning about harm reduction and safer crack use. The interviews with kit recipients were an opportunity to explore how such information had been transmitted and how learning about the safer crack use items had taken place at the time of the kit distribution and demonstrations. During the course of our analysis, it was clear that much harm reduction education information was conveyed by watching others' practices with crack use, as well as during informal interactions with other people who use crack.

As mentioned, demonstrations were an important part of the process of kit distribution; some participants were open to this mode of receiving harm reduction information. After observing a demonstration that involved the use of the brass screens, one person was receptive to trying the screens at home. Based on her comment, there had been a clear message that this was a safer approach to crack use. As a result of learning something "new," she in turn demonstrated a willingness to practice something that was less harmful.

Yeah, I know because I went home, and I was like trying it [to use screens]. You've got to always try something new, right? And if it's something that is better for me, then sure I'll do it

Following crack pipe demonstrations, learning took place by way of "hands-on" experience for many participants. During the interviews, a number of kit recipients compared their experiences with using Brillo to the use of brass screens; many, not all, indicated that they recognized that the brass screens were a safer option than was Brillo.

*The screens are good because they don't burn like the other ones, like Brillo. And the Brillo, I've had caught in my throat I don't know how many times. I've cut my fingers with it [Brillo] trying to break it apart*.

As one person stressed, being "*aware*" of health issues related to crack use had played a role in his own safer crack use practices; he indicated that this awareness was largely a result of learning about safer crack use practices through his involvement with the SCORE project. Similarly, being "aware" of the reasons for including different items was key. As an example, many participants supported the idea of supplying mouthpieces in the safer crack kits. Although the actual practices associated with these mouthpieces were not consistent, many participants were "*aware*" of their valuable role in reducing harm.

Other participants credited the impact of peers with their own learning about safer crack use; for some, information was conveyed "informally" through watching others. One woman indicated that she was using brass screens more often and remained receptive to continue to do so because of what "others" were saying.

*I've always used Brillo, but I'm finding that more people are using screens, and they're telling me, and they are showing me, "You should be doing it" which I'm doing that more often than I used to...the screens are better for your lungs, and I have emphysema, so I should be using the screens more often*.

Several participants explained how they engaged in talking about harm reduction with other people who used crack. A few participants described how they gave certain kit items to others, namely the Tip cards. A few people described the Tip cards as "*informative*" and helpful in working with others.

I found them useful in explaining to people, because I used to do outreach. And I participated in the harm reduction conference, so I'm fairly knowledgeable. So by my saying a piece of information but then having it backed up [on the card], made it invaluable, right?

One woman underscored the importance for others "*to learn*" how to use the Tip cards and take the extra time to be safer. In her interactions with others who used crack, she suggested how she emphasized that "*there are reasons why they put it in there*." Based on the interviews, it was clear that educational information and learning about harm reduction education came from different sources.

### Changing practice

Crack use practices are difficult to change. One key to the success of this project was helping persons who use crack incorporate safer practices into their lives as they saw fit. In the words of one person, the SCORE project involved "*problem solving*" that provided "*an incentive to do a safer method*."

Many participants emphasized the need for the availability of paraphernalia. If new materials such as screens were not available, people would have little choice but to use old practices.

*Well, screens aren't very available and how often does crack kits come around? I think once I got one off the street. So if they had screens available, then maybe they would be used more*.

One participant emphasized that she had decreased the sharing of pipes because pipes had become more available.

*It's safer, you're not using all broken up pipes, and we're not sharing often. I know myself, now I'm not sharing my pipes like I used to because of availability, right? And it enables you to have a new pipe almost every second day. And then you always have new hoses, new screens, so...and the thing you know we have to worry about nowadays with all these diseases that we could contact with old pipes, or sharing*.

Similarly another participant equated the availability of equipment with safety.

The benefit being that it's safer, you know, you're not using all broken-up pipes, and we're not sharing. Often, I know myself now I'm not sharing my pipes like I used to because of availability, right?

Some participants emphasized the link between having their own pipe and changes in their own use.

*Well, it's safer, instead of like people buying used ones. I used to buy used ones [pipes], and it was black [charred with use]*.

One person indicated that he was happy to have "a nice pipe" and that he no longer shared his pipe with anyone. The use of a mouthpiece was also connected to their availability.

*Everybody uses mouthpieces if they're there, pretty well, especially now people are starting to get more involved because it's a lot of sharing of pipes*.

It was clear that many of those we spoke with had developed an awareness of the harms associated with crack use practices. For example, although one participant experienced challenges using the screens, he noted that screens were safer; for this reason, he made a change in how he smoked crack.

*The screens when you use them and you heat them up, it cracks the pipe and especially in the cold, they heat up differently. The Brillo cools down kind of like that [snaps finger for effect]. You can take the screens out and wash them, or change them. People don't like the screens. As I said, I'm not a proponent of it, but no more black things spitting up, no more black tongues, um, I'm sure it will let me keep my teeth a couple of years*.

Some of the participants suggested that their new practices were becoming entrenched and were systematically changing the ways that they smoked crack.

*I mean I've been guilty of using whatever pipe was convenient and closest, whoever had whatever. And I was just lucky that I didn't catch anything from it. But now I make sure I carry my own mouthpiece with me. And if it's ever an option, I usually try not to share other people's pipes. If I absolutely cannot live without [sharing] it, then I'll have my own mouthpiece at least to put on there. And I usually carry a couple of alcohol swabs with me actually too*.

Changes were unlikely to be sustained if they were perceived to be unsatisfactory. For example, one participant's first experience using the screens was "disappointing," which influenced her plans to use screens in the future.

*I just, I didn't get anything that I was hoping to get out of it. It was really disappointing. Because I didn't do a lot of crack yesterday and to have, sometimes if you have some and you're starting fresh with something you've never tried and you use it, and you don't get what you are expecting, it's even more disappointing, so I was a little bit bummed out by that. I won't do that again because I've tried it with the screens, and every time I'm disappointed*.

Some participants who adopted screens early on indicated that they preferred the taste of using the screens. Most often, the change from using Brillo to screens was gradual for participants. Packing the pipe with the screens was a skill that required practice.

The participants who were changing their practices reflected on what they had personally found helpful in order to make changes that resulted in safer crack use, which involved incorporating what they knew about certain items, such as the screens. "*There is no such thing as safe crack, if I can minimize the damage, at least, then I'm on my way, right?*"

Some emphasized how important and helpful it was to hear the safer crack use message on a repeated basis from peers and outreach workers in order to shift personal crack use practices. One participant noted how she shared the message about safer use with others.

*Well probably the more times you're told, the more times that people are encouraging you [to use more safely]. You have the van going around telling us, now that I have a concept, I will be telling people, you know, to make a change*.

Demonstrations with pipes and screens were also beneficial in terms of changing practices. As one participant noted, this was a process that took time.

*She [outreach worker] showed me how to wrap, fold the screen, basically once she showed me that, I still didn't listen and use it. But after that, I started to, question the Brillo more. And she showed me, and you know, she just showed me what was in there [the kit] and showed me how to use the screen and that it was better for you. And I took her advice in the end, it took me awhile*.

Another interviewee noted that it took him a couple of times to learn how to use the screens properly. Thus the participants illuminate how time and repeated demonstrations are key components leading to shifting practices.

### Barriers to change

The interview data is illuminating with regards to barriers to changing established crack use practices. Access to harm reduction paraphernalia was crucial. However, many individuals articulated their resistance to changing their practice and were adamant that they were set in their ways and strongly attached to their own crack use practices. Contributing factors to such practices were the inaccurate understanding of risks (i.e., what they knew of harms), difficulty using certain paraphernalia (e.g., applying mouthpieces and screens), and crack use couched within the context of busy lives.

Some individuals indicated that they had no intention of changing how they smoked crack. As one person noted, "*I'm used to one thing, I don't change...don't even ask me because I won't change*." At the same time, there was recognition that others might be receptive to changing. "*Yeah, some people are open to change, but I'm not one of them*." Participants, particularly those with a long history of using crack, suggested that they had firmly established crack use practices and preferences. For some, personal practices were built on years of crack use. As one interviewee made clear: "*But I pretty much know like the dos and don'ts*."

One person was adamant that he would not change his crack use practice after years of doing it a certain way. In fact, he was offended by the idea that others, with presumably less experience, would show him "how to."

*Because it's almost like an insult to me because I've been smoking crack for 13 years...12 or 13 years. For somebody to demonstrate to me how to load a pipe would be disrespectful in a way*.

Another person added, "*I know all that shit already anyways, and you know, why would I need that?*"

Some people felt that they already knew harm reduction messages and that they had "*no use*" for more harm reduction information that was provided in the kit. One self described "*long-term user*" indicated that he know all the "*in's and out's*" and, along with a number of the participants, did not read the information card provided in the kit. According to another individual, this educational information was only useful for people who were learning to use crack.

*Those [cards] are for rookies, for those that don't know how to use a pipe, that's where you start learning because I'm not going to teach you. I didn't read it, didn't care because I already know how to use a pipe*.

For some older participants, a combination of accumulated knowledge and pride accompanied their longevity as people who use illegal drugs. However, their assumed knowledge hindered learning opportunities. Moreover, some of their comments raise questions of how best to communicate harm reduction information to, and take into consideration, individuals with a long history of drug use.

One significant barrier in conveying information with printed materials was noted by several participants who were not able to read the information they had received, either because they had difficulty reading or because they needed glasses. As one woman observed, a lot of people "*out on the strolls*" threw this material away because they were "*illiterate*." As one person noted, there was "too much information" on the information cards she had received.

*I don't understand that card. I don't read long things like that. Because, like some people that, they didn't understand that thing, and they couldn't read it. And some people might need glasses like me. I need glasses, but I don't wear glasses. But people get their heads smashed in and get into fights and their glasses go flying. That's why people don't wear glasses down here*.

Paraphernalia that was awkward to use was a significant obstacle. A few people noted that the mouthpiece was difficult to apply onto the pipe, which was a disincentive to using it. One person complained, "*I always seem to break the pipe when I'm putting the mouthpiece on*." It was also time-consuming when there was urgency to use.

Many people commented that, based on their experience, the screens were also problematic. This lessened the likelihood of changing how they used crack. Screens were also time-consuming to insert compared to Brillo. As a result, a number of participants highlighted the challenges related to shifting crack use practices. For some, the screens provided in the kits were far from ideal. They took time to insert, and it was thought by some people that they blocked the pipe easily. Although one interviewee found the screens to be "*perfect*" because they "*fit the pipes*" well and they did not crack easily, a number of participants stated quite simply that they preferred using Brillo because they had always used Brillo. They offered a number of reasons to support this preference. As one person explained, "*Brillo is still better than screens because it stops the oil from running through, whereas the screens, the oil runs right through it*." Not surprisingly, that participant's responses revealed that if equipment takes more time to use, is awkward to use, hinders consumption, or leads to loss of the drug, it is less likely to lead to changes in practice.

Finally, some interview data revealed the importance of conducting face-to-face interviews. For some participants, initial responses to questions about crack use practices failed to fully capture their lived experience. They also made clear the benefits of face-to-face interviews where participants have time to expand on and clarify their initial response. For example, when speaking about sharing equipment, a number of interviewees initially said they did not share. One man said: "*I'm the only one that uses my pipe*." However, he followed up by stating, "*My girlfriend is the only one that uses my pipe. So I don't bother sterilizing it*." Another interviewee also responded, "*I don't share my pipe, never have, well, I share it with my wife, but that's different, right?*" The interview process facilitated a more nuanced understanding of individuals' crack use practices and sharing of equipment with intimate partners.

## Discussion

Providing harm reduction education was extremely difficult given the context of people's lives in the DTES. At the same time, the data points to some shifts in practice that did occur for some individuals. How can we best build on the changes that did occur?

The findings suggest that availability of equipment, repeated demonstrations, watching others, peer-to-peer learning, and contact with distribution and outreach workers provide avenues for users to learn about safer crack use while obtaining harm reduction equipment. In addition, the distribution of the kits provided contact with people who use crack. The findings also suggest that there is room for improvement, such as providing better-quality screens and/or screens that are easier to use.

We found that some long-time users in our sample were not open to receiving educational information; therefore, opportunities for learning were difficult to provide. Assumed knowledge in personal crack use practices hindered learning opportunities for some people. At the same time, it is important for outreach and peer-to-peer workers to acknowledge the personal experience and expertise of those with a long history of crack use. Those individuals have much to share, and drawing on their input would enhance learning situations in the future. In addition, similar to Fraser and Valentine's 2008 study, we suggest shifting the focus from "a critical look at the behaviours' of individuals 'to a critical look at the contexts' in which individuals live" [47] (p. 12). We wish to understand the economic and social barriers that people who use crack experience and the strategies that they create to survive and to reduce harm. Some participants also made clear that illiteracy and difficulty reading (due to needing glasses) limited the usefulness of Tip cards and written harm reduction information. Thus, it is imperative that information also be provided through personal interaction, whether peer-to-peer or outreach as occurred in this project. In addition, education might be further bolstered by pictures demonstrating safer crack use.

The transmission of education and harm reduction equipment in Canada is also shaped by prohibitionist policy, which shapes the lives of people who use illegal drug (and those who do not). Partially due to the criminal status of crack, people who use the drug are depicted as criminal and deviant, rather than as individuals in need of harm reduction education and equipment, treatment, and social support. Until 2001, 95 percent of the National Drug Strategy budget was earmarked for criminal justice rather than treatment and education [48]. Changes in Canada's Drug Strategy in 2003 brought about a bit more balance, and harm reduction was included in the budget, and slightly fewer funds were allocated for law enforcement and crime control. In 2004–2005, federal Drug Strategy expenditures revealed that 73 percent of the budget went to enforcement and 3 percent on harm reduction. Coordination and research received 7 percent of the budget; prevention, 3 percent; and treatment, 14 percent [49] (p. 7). However, these small yet positive shifts were temporal. With the election of a minority conservative government in 2005, the national Drug Strategy has been restructured without consultation with public health providers, organizations such as VANDU, or drug treatment professionals. The National Drug Strategy has been renamed the "Anti"-Drug Strategy and moved from Health to Justice; the 2007 Federal Budget and Crown speech eliminated federal funding for harm reduction and grants more funding for crime control efforts. In 2007, the International Narcotics Control Board, funded by the United Nations, proclaimed that safer crack kits, mouthpiece, and pipe distribution to "chronic users" in Vancouver and the rest of Canada should be eliminated because such practices contravene existing UN drug treaties. They called on the government of Canada to eliminate these programs and to close any existing safe injection sites [50] (pp. 60, 61).

A number of local and international critics note that the International Narcotics Control Board is out of step with the rest of the UN on harm reduction and HIV/AIDS programming and aid. Critics also propose that the current federal government of Canada is out of step with provincial and municipal authorities, especially in Vancouver where established harm reduction practices have proven to be both effective and widely supported [9]. Further, in the arena of supervised injection facilities (SIF), there are concerns related to the federal government's failure to support Insite, treatment of "scientific processes and evidence," and prohibitionist policy [9,51-53]. However, a recent Supreme Court of British Columbia decision supported Insite. On May 27, 2008, Judge Pitfield ruled that closing Insite would contravene provincial access to health care and fundamental health care rights; to deny such services are an infringement of the right to life, liberty, and security of the person granted in the Canadian Charter of Rights and Freedoms [54]. Two days after the ruling, the federal government announced that they would appeal the decision.

Recent federal conflicts and initiatives create a more punitive environment than the pre-2001 Drug Strategy, especially in its rejection of positive findings stemming from harm reduction as public health initiatives. Current federal policy also makes it more difficult to envision the opening of the safer crack consumption room at Insite and support for safer crack outreach, equipment, and programs. It also challenges the vision of the enactment of social policy that assures that people's basic health, housing, and support needs are met. Nevertheless, it is interesting to speculate how such a facility would impact on harm reduction practices with regard to crack use. For example, in a well-funded and resourced safer crack consumption room, if used, sharing would be entirely eliminated in the facility (by virtue of the fact that it would be strictly forbidden in the program and prevented through observation). It is also worth noting that a SIF with a safer crack consumption room may not require a Section 56 exemption, and another facility may very likely be opened outside of Insite (especially given that Insite is already at its maximum capacity).

Critical drug research on illegal drugs illuminates how marginalization is linked to harm. Waldorf, Reinarman, and Murphy's 1991 study of individuals who used cocaine/crack found that having a "stake in conventional life" helped to keep those with the heaviest consumption from falling into negative addiction and harm [10] (p. 10). The SCORE project focused on Canada's most marginalized people who use crack in the DTES, and for many there is little access to "conventional life." Rather, similar to key ethnographic and qualitative works analyzing the social and political contexts of the lives of people who use crack [10,24,33,55,56], the lives of people who use crack in the DTES are shaped by social factors that are beyond their control: discrimination; prohibition; the role of police in enforcement, arrest, and imprisonment; lack of affordable and secure housing; inadequate health care and treatment; stigma; violence; and inadequate social and economic supports. For many participants in this sample, marginalization has been a lifetime experience.

The SCORE project is dedicated to ensuring that everyone who uses crack and lives in the DTES has access to equipment and harm reduction education that will help them to use more safely. In addition, the SCORE project exemplifies the intersection between research and practice in the community; we encourage others to consider such alternative models.

This paper highlights some of the ways that crack users are receptive to using more safely, avenues for learning, and social barriers to change. It also highlights how much further we need to go in order to provide safe and reliable access to education, information, and equipment to people who use crack, especially marginalized people as in this sample. This study suggests that harm reduction education is most successfully conveyed by watching others' practices with crack use, as well as during informal interactions with other people who use crack and repeated demonstrations of harm reduction equipment by peers and outreach workers. The findings in this paper bring to the foreground that the social context of crack users' lives in the DTES simultaneously shapes opportunities and acts as a barrier to learning. The safer crack kits made and distributed through the SCORE project in the DTES provided avenues for learning, sharing, contact, and some shifts in practice. As one participant noted, "*The kits are really useful because it gives us the sense that somebody cares*."

## Competing interests

The authors declare that they have no competing interests.

## Authors' contributions

SB is the lead author. All authors contributed to the paper and approved the final version of the paper.
